# Spatially specific mechanisms and functions of the plant circadian clock

**DOI:** 10.1093/plphys/kiac236

**Published:** 2022-05-28

**Authors:** William Davis, Motomu Endo, James C W Locke

**Affiliations:** Sainsbury Laboratory, University of Cambridge, Cambridge, UK; Graduate School of Science and Technology, Nara Institute of Science and Technology, Nara 630-0192, Japan; Sainsbury Laboratory, University of Cambridge, Cambridge, UK

## Abstract

Like many organisms, plants have evolved a genetic network, the circadian clock, to coordinate processes with day/night cycles. In plants, the clock is a pervasive regulator of development and modulates many aspects of physiology. Clock-regulated processes range from the correct timing of growth and cell division to interactions with the root microbiome. Recently developed techniques, such as single-cell time-lapse microscopy and single-cell RNA-seq, are beginning to revolutionize our understanding of this clock regulation, revealing a surprising degree of organ, tissue, and cell-type specificity. In this review, we highlight recent advances in our spatial view of the clock across the plant, both in terms of how it is regulated and how it regulates a diversity of output processes. We outline how understanding these spatially specific functions will help reveal the range of ways that the clock provides a fitness benefit for the plant.

## Teaser

Emerging tools and techniques provide insights into how the behavior and outputs of the circadian clock vary across the plant.

## Introduction

The plant circadian clock times processes throughout the day and night. To do this, the clock receives input from environmental cues, such as light and dark, and generates ∼24-h oscillation in gene expression that controls a range of output genes. In fact, transcriptomic studies have revealed that a large fraction of the Arabidopsis (*Arabidopsis thaliana*) genome is under clock control, although the exact fraction detected varies depending on experiment, from around 5.5%–37% of the Arabidopsis genome (Harmer et al., 2000; Edwards et al., 2006; Covington et al., 2008; Romanowski et al., 2020; Gardiner et al., 2021). The clock has been shown to improve the fitness of the plant; plants with a clock that matches environmental cycles outperform those that do not (Dodd et al., 2005). However, the range of mechanisms by which the plant clock can increase fitness are still being elucidated.

In this review, we will synthesize recent research examining spatially specific mechanisms and functions of the plant clock. Rather than being a single timekeeper, multiple studies are revealing specialization in regulation and function for the clock across the plant. We will only briefly describe mechanisms for local and long-distance coordination of plant clock rhythms as these have recently been extensively reviewed (Micklem and Locke, 2021; Nohales, 2021; Sorkin and Nusinow, 2021). Instead, we will focus on how the clock architecture is altered across the plant, our understanding of spatially specific functions, and what implications these have for our understanding of how the clock can increase fitness.

## Section 1: Differences in core clock structure across the plant

**Figure 1:**
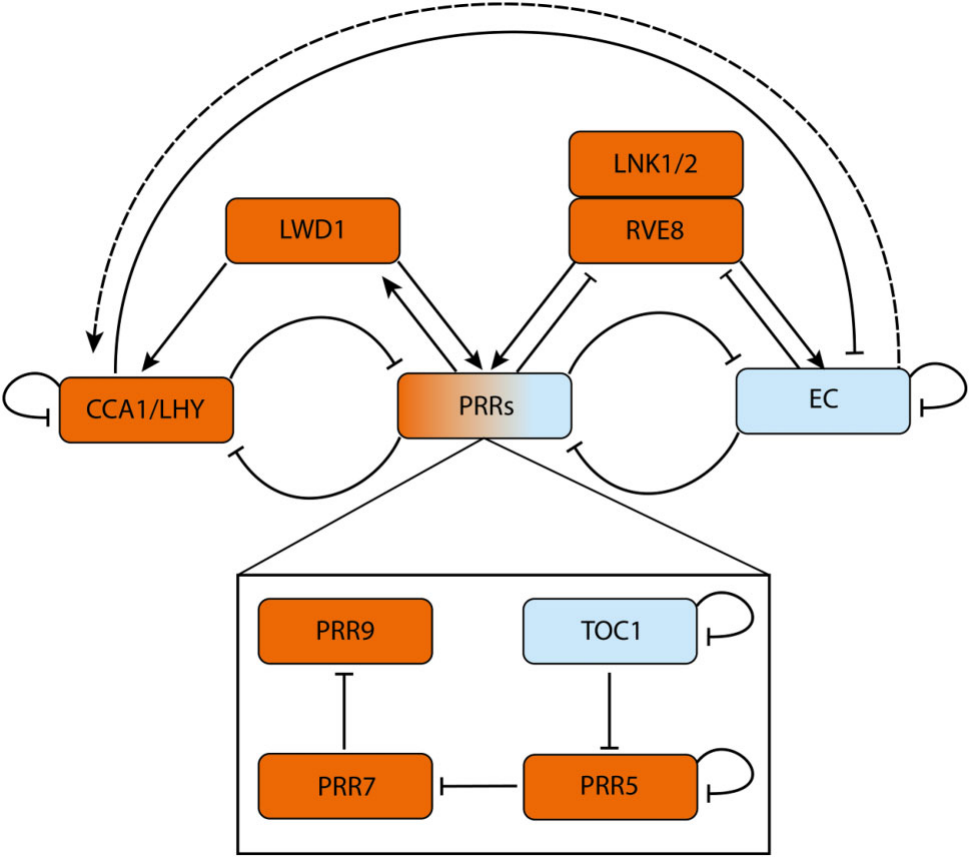
A simplified diagrammatic representation of the Arabidopsis circadian oscillator. The morning phased genes *CCA1* and *LHY* repress expression of *PRRs*, the EC and themselves. The autoinhibition of CCA1/LHY gradually derepresses expression of *PRR9*, *PRR7*, *PRR5*, and *TOC1*. The PRRs further repress *CCA1/LHY* expression. The PRRs also sequentially repress their own expression (Gendron et al., 2012; Huang et al., 2012; Nakamichi et al., 2012). At night, ELF3, ELF4, and LUX assemble into the EC. The EC represses the PRRs, thus indirectly activating CCA1/LHY (dashed line) to reset the oscillator. Further inputs to the core oscillator come from LWD1, which activates expression of (and is then upregulated by) *PRR9* and *PRR5* forming a positive feedback loop. LWD1 also acts with the TEOSINTE BRANCHED 1-CYCLOIDEA-PCF proteins TCP20 and TCP22 (data not shown) to upregulate *CCA1* expression (Wu et al., 2016). RVE8 and LNK1/2 cooperatively activate *PRRs* (*PRR9*, *PRR5*, and *TOC1*) and EC genes (*ELF4* and *LUX*) (Hsu et al., 2013), RVE8 and LNK1 are repressed by PRRs (Hsu et al., 2013) and the EC respectively (Mizuno et al., 2014). GI and ZTL (data not shown) adjust circadian rhythms. Under blue light, GI forms a complex with ZTL, stabilizing it (Kim et al., 2007). In dark conditions, ZTL then negatively regulates TOC1 and PRR5 posttranslationally by facilitating protein degradation (Más et al., 2003; Kiba et al., 2007; Pudasaini et al., 2017). Morning/afternoon and evening phased genes are colored in orange and blue respectively. Activation and repression are shown by arrowheads and flat headed lines, respectively. Interactions may be either direct (solid lines) or indirect (dashed lines).

The structure of the core clock network responsible for generating the 24-h output rhythm has been elucidated in the model plant Arabidopsis and has been shown to be dominated by repressive interactions (Figure 1). Two morning expressed genes, *CIRCADIAN CLOCK ASSOCIATED 1* (*CCA1*) and *LATE ELONGATED HYPOCOTYL* (*LHY*; Wang and Tobin, 1998), repress themselves as well as the *PSEUDO-RESPONSE REGULATORS* (*PRRs*) *PRR* family of genes, *PRR9,7,5,3*, an*d TIMING OF CAB EXPRESSION 1* (*TOC1*; Alabadí et al., 2001; Ding et al., 2007; Kamioka et al., 2016)*. TOC1* represses expression of *CCA1* and *LHY*, as well as expression of the evening complex (EC), *LUX ARRHYTHMO* (*LUX*), *EARLY FLOWERING 3* (*ELF3*), and *ELF4* (Gendron et al., 2012; Pokhilko et al., 2013). The EC goes on to repress *PRR7*, *PRR9*, and itself (Dixon et al., 2011). In addition to these repressive interactions, LIGHT-REGULATED WD1 (LWD1), REVEILLE 8 (RVE8), and NIGHT LIGHT-INDUCIBLE AND CLOCK-REGULATED (LNK) proteins provide positive interactions (Wu et al., 2008; Rawat et al., 2011; Xie et al., 2014). The experiments that originally determined this network used an average of signals from multiple plants, with each plant containing a range of organs, tissues, and cell types. Given that bulk measurements may obscure behavior at the level of single organs or tissues, this leaves an incomplete picture of the clock network across the plant. Indeed, there has long been evidence of tissue-dependent differences in the timing of the plant clock (Thain et al., 2002), and experiments at the single-cell level in Arabidopsis have revealed that clocks can display differing periods depending on the organ and their position within it (Gould et al., 2018).

To make matters more complicated, there is evidence of long distance and local coupling between circadian clocks in the plant. The shoot has been proposed to be dominant over rhythms in the root (James et al., 2008; Takahashi et al., 2015), with for example long-distance transport of ELF4 transferring temperature information from the shoot clock to the root clock (Chen et al., 2020). There is also evidence of local cell–cell coupling, which when combined with timing differences between cells can generate spatial waves of gene expression (Fukuda et al., 2007; Wenden et al., 2012; Gould et al., 2018; Greenwood et al., 2019). This coupling makes it difficult to interpret what is causing organ or tissue-dependent differences in timing in mutant studies. Is the effect of a mutation different between two tissues because the clock network topology differs between the tissues, or because the coupling between tissues or organs has been affected by the mutation? There are multiple ways that mutation of a coupling component could cause tissue-specific effects, for example, if the coupling signal travels from one tissue to the other conveying circadian information. Techniques that allow high spatial and temporal resolution measurement of clock rhythms and targeted perturbations to the clock will be required to tease apart these different possibilities (see “Section 3: Technical advances for analyzing spatial specificity of circadian rhythms” of this review).

Two different mechanisms for generating spatial differences in clock timing have been proposed. First, there can be different sensitivity to inputs to the clock across the plant. It has been hypothesized that varied sensitivities to light alter periods across the plant, which is supported by the loss of some period differences in the light sensing mutant of phytochrome B (phyB), *phyB-9* (Greenwood et al., 2019; Nimmo et al., 2020). Also, roots grown in both constant light (LL) and constant dark (DD) conditions have longer free running periods (FRPs) than shoots grown in constant light. However, the FRP of shoots in DD is longer than in LL and more similar to the FRP of roots (Bordage et al., 2016). The lengthened shoot period in DD is observed even when plants are grown with sucrose present, eliminating the possibility of differences in photosynthetic outputs contributing to tissue-specific periods. This may indicate that the FRP of shoots is more sensitive to light inputs than the FRP of roots.

The other mechanism to generate spatial differences in clock timing is differences in the core clock network architecture. Recent work has updated our understanding of how the clock network might differ across the plant. This work has uncovered important roles for the PRRs and EC in generating tissue-specific differences between the root and the shoot clocks. Abolishing expression of *PRR7* or *PRR9* and *PRR7* (but not *PRR9* alone) decreases the period difference between the root and shoot (Li et al., 2020). The input of PRR7 into shoot-specific circadian network behavior may be partly mediated through *CCA1*. The nonfunctional *prr7-11* mutant displays a sharp increase in *CCA1* expression at the transition from dark to light, as well as a sharp decrease at the transition from light to dark, in shoots but not roots (Nimmo and Laird, 2021). A reduced robustness of *CCA1* oscillations in *prr7-11* shoots but not roots was also observed. Nimmo and Laird do not observe abrupt shifts in *GIGANTEA* (*GI*) expression between light and dark in a *prr7-3* mutant, which contains the same T-DNA insertion as *prr7-11*, but the *prr7-3* mutant did abolish the phase difference in *GI* expression between roots and shoots. These results support the proposal of Webb et al. (2019) that *prr7* mutations affect the plasticity of the clock. Mutants in different components of the EC also show differential clock rhythmicity between root and shoot. For example, the clock in roots but not shoots is arrhythmic in *lux-4* mutants and the reverse is true for *elf3-2* plants (Nimmo et al., 2020), indicating that each component is more important for the clock in one tissue compared to the other. It is thus clear that further work is required to unravel how the clock structure varies between roots and shoots in plants.

An approach to clarify our understanding of organ-specific clock function was taken by using luciferase imaging of a *CCA1::LUC* reporter in individual root or leaf-derived cells in cell culture (Nakamura and Oyama, 2021). This builds on previous research examining clock rhythms in cell culture and in excised parts of the plant (e.g. Nakamichi et al., 2003; Takahashi et al., 2015; Nakamura and Oyama, 2018). Nakamura and Oyama found that at the single-cell level both shoot and root cell types displayed rhythms that were temperature compensated (meaning the periods of the oscillations remain unchanged over a range of temperatures), although the amplitude of root-derived rhythms damped more than shoot-derived rhythms under constant darkness. Roots and aerial tissues also differ in their entrainment characteristics. Interestingly, isolated shoot cells entrain less accurately to light dark cycles than isolated root cells. However, dense cultures of shoot cells showed more accurate rhythms than isolated shoot cells, suggesting that entrainment accuracy might depend on both cell type and cell density in plant organs. This fits with previous results showing that denser cell cultures exhibit more robust rhythms, potentially due to cell–cell coupling of rhythms (Takahashi et al., 2015).

Insights into tissue-specific clock behavior may also be informed from studies in other plant species. In contrast to Arabidopsis, the period of circadian rhythms in the model legume *Medicago truncatula* is shorter in roots than in shoots (Wang et al., 2021a). As the phase of the clock in *M. truncatula* is earlier in the roots than the shoots, this is inconsistent with a model where the shoot drives rhythms in the root. It will be interesting to examine species-specific mechanisms of clock co-ordination. Duckweed (*Lemna* spp.) fronds have also proved to be an excellent model system to examine single-cell clock properties and clock coupling (Muranaka and Oyama, 2016). Recently, local cell–cell coupling was shown to coordinate the *Lemna minor* frond clock and to drive circadian waves of clock gene expression during the proliferation of the fronds (Ueno et al., 2021). Coupling between cells decreased the period of cellular rhythms, generating a centrifugal pattern of periods in proliferating fronds. Interestingly, the strength of the coupling decreased with the age of the frond. *L. minor* fronds that were never given an entrainment signal, only grown in constant light, generated spontaneously organized waves similar to that observed in Arabidopsis plants grown without entrainment (Wenden et al., 2012; Greenwood et al., 2019). Further studies in other plant species on how plant tissue structure can shape spatial patterns in clock gene expression will extend our knowledge of tissue-specific clock behavior beyond model organisms.

## Section 2: Spatially specific functions of the clock

**Figure 2:**
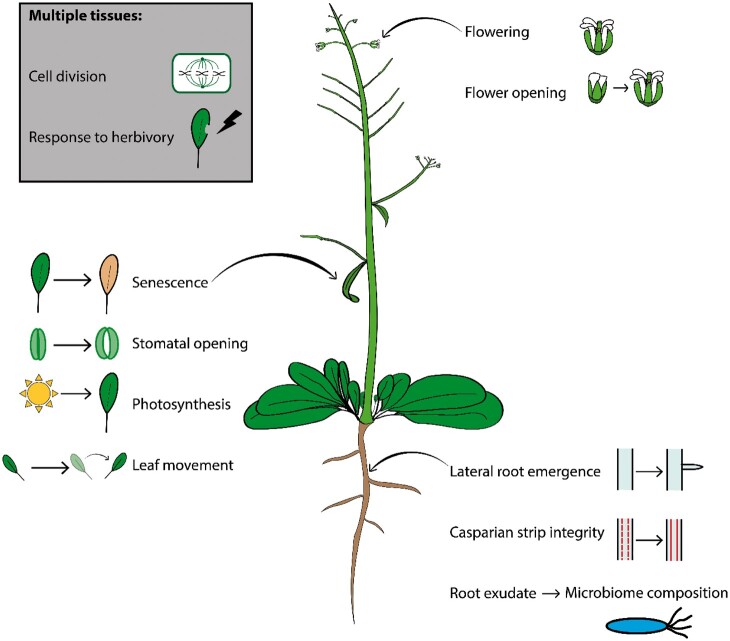
Spatially specific functions of the plant circadian clock. The clock coordinates biological processes across the plant, in individual organs, tissues, and cell types. These include senescence (Song et al., 2018), stomatal opening, photosynthesis (Dodd et al., 2005) and rhythmic movement in leaves (Kong et al., 2020), lateral root development (Voß et al., 2015), root exudate composition (Lu et al., 2021), and CS integrity in roots (Durr et al., 2021), as well as flowering (Hicks et al., 2001) and flower opening (Muroya et al., 2021). Root-specific clock functions can also feed into microbiome composition (Durr et al., 2021). The clock regulates processes common to multiple tissues such as cell division and response to herbivory.

We now describe recent progress in revealing the mechanisms underlying spatially specific functions of the circadian clock in plants. The clock has been shown to modulate a whole host of processes across the plant, including photosynthesis (Dodd et al., 2005), senescence (Song et al., 2018), leaf movement (Kong et al., 2020), flower opening (Muroya et al., 2021), and lateral root development (Voß et al., 2015; see Figure 2). We will focus on recent interesting findings concerning clock control of growth and cell division, the role of clock components in temperature perception, and the role of the clock in modulating inter-species interactions.

### Clock modulation of growth and cell division

Cell division in plants is primarily restricted to meristems, and cell elongation can be activated or inhibited independently in different tissues. Growth is therefore a spatially specific process that can be optimally timed to take advantage of the diel cycles of resource availability. Unsurprisingly, plant growth is, therefore, gated by the circadian clock to specific times of day. For example, root elongation rate peaks at dawn, showing circadian rhythms which are abolished under constant conditions in *elf3-1* mutants (Yazdanbakhsh et al., 2011). The clock also rephases in lateral roots during their initiation. Disruptions to the clock lead to defects in lateral root development, possibly due to circadian gating in auxin signaling required to generate hydraulic conditions that facilitate lateral root emergence (Voß et al., 2015). Recent research has shown how multiple clock components repress the PHYTOCHROME-INTERACTING FACTOR (PIF) family of transcription factors, which promote growth, to restrict hypocotyl elongation to specific times of day. The clock is key to causing hypocotyl elongation to peak at the end of the night under short days (8-h light/16-h dark; Nozue et al., 2007). PIF activity during the day is repressed by light mediated protein degradation from the photoreceptor phyB and transcriptional regulation from the PRR clock components (Martín et al., 2018; Zhang et al., 2020; Yan et al., 2021), whilst GI and the EC repress *PIF* expression during the evening and early night (Anwer et al., 2020; Park et al., 2020). Under short days, but not long days, clock repression of *PIF* expression is released before dawn, providing a window in which PIF activity promotes hypocotyl elongation.

As well as gating growth to specific times of day through the regulation of PIFs, the clock has also been shown to have a direct input into cell division, by controlling the length of S-phase in Arabidopsis (Fung-Uceda et al., 2018). The core clock protein TOC1 binds to and represses the expression of *CELL DIVISION CONTROL 6*, a regulator of DNA replication during S-phase involved in establishing the DNA replication complex. TOC1 was also found to indirectly regulate a host of other cell cycle regulators, including upregulating *CYCLIN D3;3*. The effects of this regulation appear wide ranging, with TOC1-OX plants having dwarf phenotypes, a reduced cell division rate in developing leaves and impeded progression of *Agrobacterium tumefaciens* induced tumors. In the future, it will be interesting to compare the extent of clock control of cell division in different organs, for example shoot versus root, and to examine whether the clock causes cell division to be promoted or repressed at particular times of day or night as is found in cyanobacteria (Mori et al., 1996; Martins et al., 2018). It will also be important to test whether tissue-specific clock properties affect the clock’s modulation of cell division (e.g. does the increased expression of *TOC1* in vascular tissues confer specific cell division properties to this tissue? (Endo et al., 2014).

The regulation of growth and cell division by the clock likely often interacts with environmentally responsive pathways. A prime example of the interaction of environmental stress, the clock, and growth, is that of shade avoidance. The shade avoidance response represents a tissue-specific input to development, as it is sensed in aerial tissues. Under prolonged shade, plants respond with a suite of morphological changes, including elongated petioles, increased hypocotyl elongation, and early flowering (reviewed in Wang et al., 2020b). Interestingly, the clock components PRR5 and 7 have been shown to repress the hypocotyl elongation response in Arabidopsis under shading through their interactions with PIFs, preventing the over elongation of stems (Zhang et al., 2020). Under deeper canopy shade, any hypocotyl elongation can be detrimental to the plant due to the extremely limited resources. Recently, it has been shown that the activation of the photoreceptor phytochrome A under shaded conditions promotes increased expression of *TOC1*, *PRR7*, *ELF3*, and *ELF4* (Fraser et al., 2021). As discussed above, all these components repress hypocotyl elongation, showing how environmental stress and the clock can be integrated to modulate development. Interestingly, the balance between evening and morning-expressed clock genes was also observed to be altered under shading in the grass sorghum (*Sorghum bicolor*; Kebrom et al., 2020), suggesting that interactions between the clock, light quality, and growth, could be conserved.

### Clock mediated temperature perception in plants

Whilst mutations to components of the core circadian oscillator affect the period, phase, and amplitude of circadian rhythms, clock mutants are often only arrhythmic without entrainment cues (e.g. light or temperature). Despite this, even when rhythms are maintained under light–dark cycles, clock mutants have distinct phenotypes—for example, loss of function mutations in ELF3 cause early flowering (Hicks et al., 2001; Fukazawa et al., 2021), whereas those in GI delay flowering (Gould et al., 2006). Intriguingly, clock mutants that are arrhythmic in light–dark cycles, for example, the *prr9 prr7 prr5* (*prr975*; Nakamichi et al., 2009) or *lux-1* (Hazen et al., 2005) mutants, also have contrasting phenotypes, with *prr975* and *lux-1* conferring late and early flowering respectively. This is an indication that the functions of the circadian clock go beyond time keeping and is consistent with the differences in transcriptional targets of individual clock components.

The functional effects of different core clock outputs can make it difficult to interpret whether a phenotype in a clock mutant is due to changes in clock timing or due to the missing functionality of the component itself. This is particularly the case for temperature perception, given that the core clock component ELF3 has recently been shown to be a direct sensor of temperature (Jung et al., 2020). ELF3 had been previously shown to play a role in the thermosensory regulation of hypocotyl elongation in Arabidopsis, as at 27 degrees it no longer represses hypocotyl elongation (Box et al., 2015). In this work, the authors used confocal microscopy to demonstrate that an ELF3–GFP fusion protein undergoes a phase transition under higher temperatures to form inactive speckles in individual cells. This phase transition is reversible and is because ELF3 contains a polyglutamine (polyQ) repeat that is embedded within a predicted prion domain (PrD). The authors show that this PrD is required for thermosensory regulation of hypocotyl elongation, as well as the formation of speckles of ELF3–GFP at higher temperatures. The finding that ELF3 stability is temperature dependent has implications for clock timing that require further investigation. Intriguingly, the authors propose that ELF4 can stabilize ELF3 at higher temperatures, with ELF4 binding to a region adjacent to the PrD. This could be of particular interest, as ELF4 has been recently shown to transmit circadian temperature information from the shoot to the root in plants (Chen et al., 2020). Chen et al. use confocal microscopy and grafting experiments to show that ELF4 moves from shoots to regulate rhythms in roots in a temperature-dependent manner. Low temperatures favor ELF4 mobility, causing a slower paced root clock, whilst higher temperatures decrease movement, leading to a faster clock. In the future, understanding the interactions between the clock and temperature will allow us to integrate our understanding of ELF4 shoot to root movement with the role of ELF3 as a thermosensor.

Further evidence of the need for more study comes from the recent work by Ronald et al. (2021), who do not observe speckles in their 35S::ELF3-YFP construct at elevated temperatures. Instead, they observe foci of ELF3-YFP in individual cells at ambient temperatures that disappear at higher temperatures. These foci could be sites where the EC binds to DNA and represses gene expression. They propose the differences between the studies could be due to differences in the time of day of the application of the temperature elevation, as Ronald et al. apply their temperature elevation at dusk when ELF3 interacts with other EC components, but ELF3 can also co-localize with morning phased proteins such as phyB and TANDEM ZINC-FINGER PLUS3.

### Clock modulation of inter-species interactions

In nature, plants share their environment with a host of species. Inter-species interactions play a vital role in plant function. Most obviously many plants depend on pollinators, driving the evolution of a host of attraction strategies such as nutrient rewards (in the form of nectar) and release of appealing scents (Wragg and Johnson, 2011; Parachnowitsch et al., 2019). Because pollinators are typically active at specific times during the day, plants must carefully time flower-specific processes to avoid wasting resources, necessitating regulation from the circadian clock. Flower-specific functions of the circadian clock in mediating plant–pollinator interactions have been recently reviewed (Bloch et al., 2017). It is also clear that plants can have complex and often beneficial relationships with the root microbiome. For instance, a strain of *Bacillus subtilis* can upregulate nitrogen reductase activity in Arabidopsis, which leads to a growth advantage (Lee et al., 2020). Plants can support microorganisms through the root-mediated release of organic molecules, including amino acids (Lesuffleur et al., 2007), sugars (Lugtenberg et al., 1999), and organic acids (Sandnes et al., 2005). The influence of circadian rhythm on metabolism and transport of metabolites is well known (Yazdanbakhsh et al., 2011; Zhu et al., 2018; Augustijn et al., 2019; Cervela-Cardona et al., 2021) and extends to root exudate composition. Although earlier studies suggested that only seven phytochemicals from Arabidopsis show diurnal rhythms in soil accumulation (Badri et al., 2010), more recent work has identified up to 50 in wild-type plants, but a reduced number in *TOC1* (*toc1-101*) and *CCA1* (*cca1-1*) mutants (45 and 39, respectively; Lu et al., 2021). Soil conditioned with *TOC1* and *ZTL* mutant plants had a reduced root microbiome diversity, and wild-type plants grown in *TOC1/ZTL* mutant conditioned soil showed reduced biomass compared to plants grown in wild-type conditioned soils (Hubbard et al., 2018).

These studies demonstrate that diurnal rhythms and circadian gene expression exert influence over root microbiome and exudate composition, with downstream consequences for fitness, but do not assess tissue-specific clock functions. Observations from Lu et al. and Hubbard et al. could be explained by inputs from multiple different tissues, for example, by changes to photosynthetic output, metabolite transport from shoot to root, secondary metabolite biosynthesis, and/or transport within the root (Butler et al., 2004). One root-specific clock output that can modulate the root microbiome is Casparian strip (CS) function. The CS acts as a physical barrier in the endodermis, regulating export of water and various solutes, including important components of root exudates such as glucosinolates (Xu et al., 2017). Loss of function mutations in the clock regulatory gene *TIME FOR COFFEE* disrupt the formation of the CS, altering accumulation of many bacterial species in the rhizosphere (Durr et al., 2021). Interestingly, Durr et al. (2021) find no effect of *TOC1* knockdown by RNAi on CS integrity, suggesting that clock-dependent effects on the root microbiome act through specific pathways.

In the model legume *M.truncatula*, root nodules (specialized structures which accommodate nitrogen-fixing bacteria) have tissue-specific circadian clocks: *MtLHY*, *MtTOC1a*, *MtPRR5/9*, and *MtLUX* are all rhythmically expressed in roots and shoots, but in nodules only *MtLHY* is rhythmically expressed (Kong et al., 2020). Plants with disrupted MtLHY function that were inoculated with *Sinorhizobium meliloti* (a species of nitrogen-fixing bacteria) had fewer nodules and reduced fresh shoot weight than wild-type inoculated plants (Kong et al., 2020). Kong et al. also show that nodulation feeds back into the circadian rhythms of aerial tissues through altered nitrogen assimilation, causing a phase advance.

Beyond the rhizosphere, a diverse community of microbes present on the aerial tissues of the plant (known as the phyllosphere) also modulate plant health and nutrition. Foliar spray of several bacterial species onto maize (*Zea mays* L.) increases plant dry weight (Abadi et al., 2020). To date, no studies have comprehensively investigated the effect of clock mutations on composition of the phyllosphere microbiome. Further work is also needed to understand the extent of clock modulation of root exudate and root microbiome composition, for example, by testing for rhythms in microbiome composition under constant environmental conditions. Furthermore, future work could also uncover root-specific clock functions in microbiome organization by specifically disrupting the expression of clock genes in root tissue only, or through micrografting experiments.

Clock-mediated processes in specific tissues also modulate interactions with herbivores. Because herbivore activity also oscillates across the diel cycle (Zhang et al., 2021a), temporal coordination of response to herbivory can increase fitness (Goodspeed et al., 2012, 2013). Rhythmic expression of genes regulating indole glucosinolate biosynthesis are attenuated in *cca1-11 lhy-21* Arabidopsis plants, rendering them more susceptible to feeding from aphids (Lei et al., 2019; Lei and Zhu-Salzman, 2021). Interestingly, the clock may differentially regulate synthesis of defense-related metabolites across the plant. The metabolite nicotine mediates defense against herbivory in *Nicotiana attenuata* and, although synthesized specifically in roots, is transported to all tissues in response to wounding (Li et al., 2018). Silencing of the clock protein ZTL downregulates nicotine accumulation, subsequently increasing susceptibility to a generalist herbivore *Spodoptera littoralis*. Furthermore, the clock has roles in balancing resource allocation to defense metabolites. TOC1 silenced plants show decreased accumulation of the defense metabolite dicaffeoylspermidine and increased nicotine accumulation (Valim et al., 2020). The increase in nicotine accumulation depends on functional ethylene signaling. Ethylene is induced during herbivore feeding and modulates defense responses in conjunction with jasmonate signaling. Blocking ethylene signaling reverses differences in nicotine accumulation between wild-type and TOC1 silenced *N. attenuata* plants (Valim et al., 2020). In Arabidopsis, loss of function mutants in another clock gene *XAP5 CIRCADIAN TIMEKEEPER* show specific defects in response to ethylene in aerial tissues, but not in roots (Ellison et al., 2011). These works reveal that the clock can modulate defense against herbivory through multiple pathways, and that tissue-specific analyses are required to understand these mechanisms.

## Section 3: Technical advances for analyzing spatial specificity of circadian rhythms

**Figure 3:**
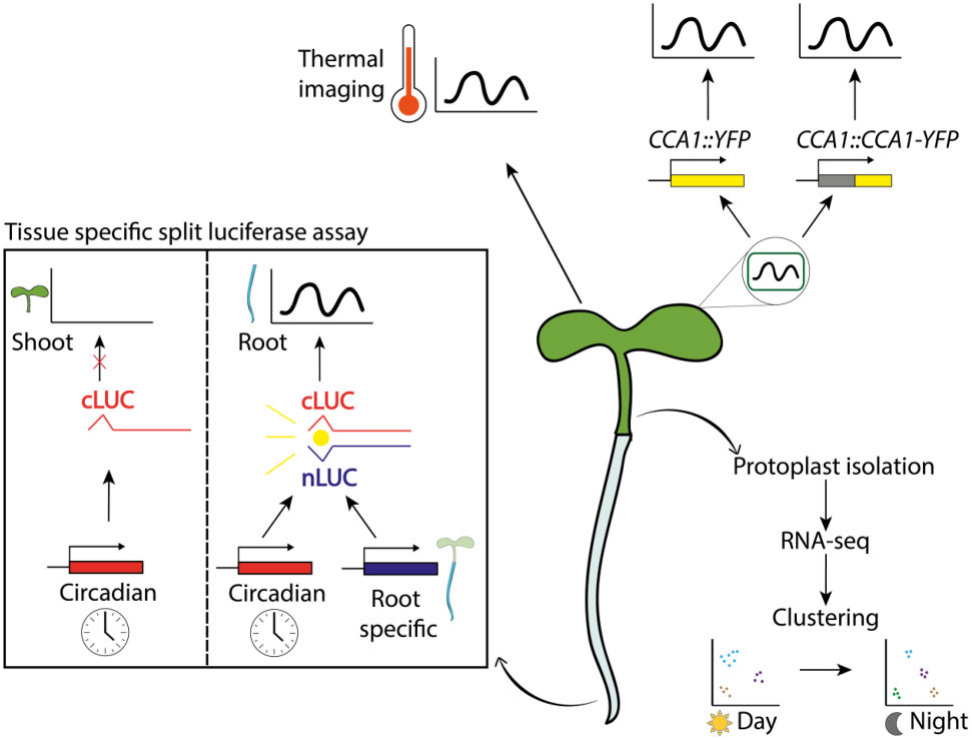
Technical advances for analyzing spatially specific circadian rhythms. Tissue-specific circadian rhythms can be measured using thermal imaging (top left) (Dakhiya and Green, 2019) and luciferase imaging. Spatial studies of clock gene expression can be enhanced by tissue-specific split luciferase assays (box, left). One construct, expressed under a circadian promoter, comprises the C-terminal half of luciferase fused to the C-terminus of A-Fos. A second construct consisting of the N-terminal half of luciferase fused to the bZIP domain of c-Jun is constitutively expressed under a tissue-specific promoter (either directly or indirectly through a UAS promoter transactivated by enhancer trap-driven expression of GAL4; Román et al., 2020). When both constructs are expressed together, the A-Fos and c-Jun domains facilitate dimerization, bringing the two halves of luciferase into close proximity to reconstitute enzymatic activity (Endo et al., 2014), generating luminescence from circadian rhythms only in a target tissue (e.g. root). Single-cell clock functions can be studied with transcriptional (e.g. CCA1::YFP) or translational (e.g. CCA1::CCA1-YFP) fluorescent reporters (top right) and single-cell RNA-seq (bottom right). Clustering of single-cell transcriptomes at separate time points provides extra information on circadian regulated functions in specific cell types (Apelt et al., 2021).

As demonstrated in the previous sections, determining organ or tissue-specific behavior and functions of circadian clocks poses unique challenges: the cycling expression of clock genes necessitates that measurements are taken over long time periods with high temporal resolution. Here, we discuss recent advances in methods for studying spatially specific clock networks, highlighting their advantages and limitations (Figure 3).

Micrografting is a simple method that can introduce organ-specific perturbations in clock gene expression, whereby tissue from a wild-type seedling is attached to tissue from a seedling with a differing genotype, generating chimeric plants (Turnbull et al., 2002; Bainbridge et al., 2014). Micrografts using clock mutant and wild-type seedlings, for example, have been used to investigate clock coupling signals between roots and shoots (Takahashi et al., 2015; Chen et al., 2020). Although advances have been made in the range of organs and tissues amenable to grafting, grafting approaches remain infeasible for studying many tissue types, such as the vasculature. An alternative solution for tissue-specific genetic manipulation of the clock in these cases may come from CRISPR tissue-specific knockouts, in which a Cas-9 nuclease and gRNA targeting a gene of interest are expressed using a tissue-specific promoter (Decaestecker et al., 2019).

One of the most commonly used techniques in studying spatial coordination of the plant clock is luciferase imaging. The promoter of a clock/output gene is used to drive expression of a luciferase enzyme (such as firefly luciferase; Ow et al., 1986) which, in the presence of cofactors (Mg^2+^, oxygen, and ATP) and the substrate D-luciferin, produces a molecule which decays to produce light (Green and McElroy, 1956). Detection of these light signals by a camera allows for time-lapse imaging of expression patterns, typically within single or multiple seedlings. This approach was used by Thain et al. (2002) to demonstrate that expression of *CHALCONE SYNTHASE* (*CHS*), a gene expressed mainly in epidermal cells of aerial tissue, oscillated with a longer period than the predominantly mesophyll-specific *CHLOROPHYLL A/B BINDING PROTEIN* (*CAB*). Though Thain et al.’s luciferase imaging gave tissue-specific information, this was only due to the tissue-specific expression patterns of *CHS* and *CAB*. Core clock genes are expressed across multiple tissue types (Takahashi et al., 2015). Tissue-specific studies of core clock expression can be enhanced by using split luciferase assays: here, two separate halves of luciferase (one N-terminal and one C-terminal half) are fused to two proteins which strongly interact with one another (c-Jun and A-Fos, respectively; Olive et al., 1997). One protein is expressed using a tissue-specific promoter and the other protein under a circadian promoter. The activity of the luciferase enzyme is reconstituted only in parts of the plant where both constructs are expressed. Consequently, clock promoter activity can be assayed over time in specific tissues (Endo et al., 2014). Enhancer trap split-luciferase assays further refine this method by expressing the c-Jun-nano luciferase (nLUC) fusion under a UAS promoter, which is activated by binding of GAL4. Expressing *GAL4* using enhancer trap systems, which consists of previously characterized lines that drive strong tissue-specific expression under a single enhancer (Shima et al., 2016), avoids the need to use full promoters, which can introduce unwanted regulatory effects (Román et al., 2020). However, spatial position of cells can affect clock gene expression even within the same tissue (Fukuda et al., 2007; Wenden et al., 2012; Gould et al., 2018). Increasing the resolution of luciferase imaging, therefore, remains a priority. Toward this goal, protocols for luciferase imaging with sub-tissue resolution have been described for Arabidopsis (Furuhata et al., 2020; Greenwood et al., 2022) and other plants, including soybean (*Glycine max* (L.) Merr.; Xie et al., 2022) and duckweed (Muranaka et al., 2013; Muranaka and Oyama, 2020).

Though challenging, luciferase imaging has been performed at single-cell resolution in the duckweed *Lemna gibba*. This imaging uncovered heterogeneity in the circadian period between individual cells under constant dark and constant light conditions (Muranaka and Oyama, 2016). More recently, Watanabe et al. introduced a dual color luciferase reporter system into *L.gibba*: using two separate luciferases which emit light at different wavelengths and a filter to separate detection of these two wavelengths, they visualized expression of a clock reporter (*AtCCA1*) and a clock output simultaneously in single cells over time (Watanabe et al., 2021). This work is of exceptional interest: separating the influences of spatially heterogeneous clock network behavior and tissue specificity in regulation of clock output expression could further the understanding of tissue-specific functions for the clock. However, the flat, disc-like structure of *L. gibba* facilitates single-cell luciferase imaging studies. Plants with deeper and more complex tissues (e.g. Arabidopsis) would be more difficult to image at a single-cell level using luciferase.

Most clock studies using luciferase imaging focus on transcriptional clock dynamics. However, it is also possible to study protein–protein interactions using a split luciferase assay. By splitting the two functional domains of luciferase between two proteins of interest, luciferase function (and thus light output) can be reconstituted only when the two proteins interact (Fujikawa and Kato, 2007). Whilst this method is limited by potential disruption to endogenous interactions by the addition of the luciferase domains to the proteins of interest, the recent development of a Nluc system may address this issue given its smaller size (Urquiza-García and Millar, 2019; Wang et al., 2020a). Protein–protein interactions, such as those between ELF3, ELF4, and LUX in the EC, are critical in clock function. Studying the dynamic assembly and disassembly of protein complexes such as the EC in a tissue-specific manner can give further insight into tissue-specific behavior of the clock (Li et al., 2020).

Unequal penetration of luciferin into specific tissues may limit studies of tissue-specific clocks. Therefore, methods to produce endogenous luminescence are needed. By introducing enzymes from fungi bioluminescence pathways that convert caffeic acid—an intermediate metabolite in the synthesis of lignin—into luciferin, stable auto-luminescence has been generated in several plant species (Khakhar et al., 2020; Mitiouchkina et al., 2020). Although this approach may require ectopic expression of enzymes to produce caffeic acid in tissues where endogenous abundance is poor, auto-luminescence could be combined with tissue-specific split luciferase assays (as in Román et al., 2020.) to study the clock in tissues with poor coverage from luciferin.

Confocal fluorescence microscopy can also allow the tracking of single-cell circadian rhythms (Yakir et al., 2011; Takahashi et al., 2015; Gould et al., 2018). Confocal microscopy has the advantages of allowing deeper tissue measurements of single-cell rhythms. For example, tracking of the nuclear levels of a fluorescent clock reporter, CCA1::CCA1-YFP, across Arabidopsis seedlings revealed tissue-specific differences in timing, including faster rhythms in multiple cell layers of the root tip (Gould et al., 2018). Confocal microscopy can also allow the examination of localization of clock proteins in specific tissues. For example, the use of fluorescent fusion proteins have allowed visualization of ELF4 protein movements from shoot to root, independent of *ELF4* mRNA location (Chen et al., 2020). Further, fluorescence microscopy approaches can visualize additional biological processes compared to luciferase imaging. By tagging the blue light receptor cryptochrome 2 (cry2) with YFP, Wang et al. observed dynamic formation of cry2 photobodies in single cells through liquid–liquid phase separation in response to blue light (Wang et al., 2021b). In combination with the RNA adenosine methylase MTA, these photobodies regulated stability of *CCA1* and *LHY* transcripts through adenosine methylation, subsequently shortening circadian period. Regulation of circadian clocks from miRNAs has also been proposed in plants (Feng et al., 2020). Assays using fluorescent reporters engineered with miRNA binding sites have been used to explore dynamics in miRNA activity in single mammalian cells (Ando et al., 2017). Thus, fluorescence microscopy could assess the influence of miRNA activity on circadian rhythms in specific cell types in plants. Fluorescence microscopy can be limited by focus drift—gradual unintended shifts in the focal plane—and, due to the scanning of the sample with a laser, photobleaching: light induced damage to a fluorophore which reduces its fluorescence over time. Techniques are being developed to limit these issues. In particular, light sheet microscopy and spinning disc microscopy offer alternatives to conventional confocal microscopy that reduce issues with bleaching of the sample (Berthet and Maizel, 2016; Komis et al., 2018).

Several studies have examined clock expression in specific parts of the plant using a combination of tissue dissection and RNA-seq/microarrays (Endo et al., 2014; Xing et al., 2019; Cramer et al., 2020; Zhang et al., 2021b) or RT-qPCR (Shimizu et al., 2015; Bordage et al., 2016; Li et al., 2020). RNA-seq in particular allows global measurement of transcripts in a single sample. This is an advantage compared to luciferase and confocal measurements, which typically only allow 1–2 clock processes to be tracked at a time. However, as RNA-seq measurements are destructive, they do not allow the tracking of the same plant’s gene expression over time. Single tissue studies in gene expression conducted through laser capture and microdissection have given valuable insights into the clock (Endo et al., 2014), although these preclude the analysis of rare, difficult to separate tissue types (e.g. the quiescent center in roots). Excitingly, single-cell RNA-seq (scRNA-seq) technologies offer a method to examine the clock in these rare cell types, as well as a method to examine the clock in single cells during development. Recently, a role for the circadian clock in vascular cell differentiation was revealed using scRNA-seq that was carried out on cells obtained using glass capillaries to avoid the stress of protoplasting (Torii et al., 2019). The release of a scRNA-seq dataset of Arabidopsis aerial tissues and roots harvested at specific time points within the diel cycle (Apelt et al., 2021) also represents a promising future direction for analyzing clock regulation of transcriptomes at the single-cell level in specific cell types.

The chromatin landscape in Arabidopsis is dynamic, circadian regulated, and differs between tissues and cell types (Farinas and Mas, 2011; Baerenfaller et al., 2016; Tong et al., 2020; Tian et al., 2021). Furthermore, chromatin organization has a role in regulating expression of clock genes (reviewed in Barneche et al., 2014; Du et al., 2019). Therefore, studying chromatin organization over time could provide insight into tissue-specific behavior and functions of the clock. Assay for transposase accessible chromatin sequencing (ATAC-seq) represents a rapidly advancing technology that could fulfill this purpose. In ATAC-seq, the transposase enzyme Tn5 introduces sequencing adaptors into the genome. Because these adaptors are more likely to be inserted where chromatin is accessible, sequencing after Tn5 activity reveals information on chromatin structure (Buenrostro et al., 2015). An atlas of single nucleus ATAC-seq (sNucATAC-seq) data from Arabidopsis roots has revealed regions of accessible chromatin associated with cell type-specific gene expression (Farmer et al., 2021). By performing a time course sNucATAC-seq under constant environmental conditions, tissue-specific circadian regulation of chromatin could be uncovered. This would represent a substantial technical advance, as current studies in diel rhythms of chromatin state depend on bulk studies using techniques such as chromatin immunoprecipitation sequencing (ChIP-seq; Song et al., 2019).

ATAC-seq is limited to assessing only chromatin accessibility. Deciphering the role of chromatin structure in tissue-specific clock functions will require understanding of how clock transcription factors and clock-regulated chromatin remodeling proteins differentially bind DNA in specific cell types. ChIP-seq has been used to assess DNA-binding targets for most clock transcription factors (Gendron et al., 2012; Nakamichi et al., 2012; Liu et al., 2013, 2016; Ezer et al., 2017; Adams et al., 2018), but these experiments have all been conducted using bulk measurements of seedlings or tissues, and thus have low spatial resolution. Although single-cell ChIP-seq has been achieved in mammalian cell lines (Grosselin et al., 2019), this is currently not yet possible for plants.

Finally, thermal imaging is a recently developed method for tracking circadian rhythms. By using a highly sensitive detector, it was demonstrated that the surface temperature of different organs (particularly leaf versus flower) displayed distinct circadian rhythms (Dakhiya and Green, 2019). This method was also able to assess tissue-specific clocks in other plants, and at later developmental stages. In contrast, high-resolution luciferase imaging and time-lapse confocal microscopy are typically limited to seedlings, and require the generation of reporter lines. However, their approach was limited only to aerial tissues. Combining the protocol with a method to assay the root clock could facilitate studies in organ-specific clock properties in a wider range of plant species.

## Concluding remarks

In this review, we have updated on recent progress in understanding spatially specific mechanisms and functions of the plant circadian clock. It is an exciting time in plant circadian biology, as the increasing availability of single-cell or single-tissue techniques for assaying the clock are helping to reveal the range of tissue and cell-type-specific clock mechanisms across the plant. In the future, we can look forward to building a comprehensive understanding of how the clock integrates entraining signals from across the plant and coordinates such a range of outputs (“Outstanding Questions” box). This will help us understand how the clock provides a fitness benefit for the plant through its many interactions with key cellular processes.

ADVANCES

- Differences between the shoot and root clock network have been elucidated.
- Mechanisms for clock control of growth and cell division timing have been proposed.
- A role for the clock in modulating microbiome interactions has been proposed.
- Recently developed luciferase and confocal imaging techniques allow tissue and cell type-specific measurement of clock rhythms.

OUTSTANDING QUESTIONS

- What is the extent of cell-type-specific clock regulation?
- How many clock coupling components are there?
- How do spatially specific circadian functions coordinate to modulate inter-species interactions?
- Do the characteristics of tissue-specific clock networks change during development?

## Notes

M.E. and J.C.W.L. made the funding acquisition and resources; W.D., M.E., and J.C.W.L. conceptualized the study, wrote the original draft, and reviewed and edited the manuscript.

The author responsible for distribution of materials integral to the findings presented in this article in accordance with the policy described in the Instructions for Authors (https://academic.oup.com/plphys/pages/general-instructions) is James C.W. Locke (James.Locke@slcu.cam.ac.uk).
